# Enhancing textural properties in plant-based meat alternatives: The impact of hydrocolloids and salts on soy protein-based products

**DOI:** 10.1016/j.crfs.2023.100571

**Published:** 2023-08-21

**Authors:** Somayeh Taghian Dinani, Yunyu Zhang, Bongkosh Vardhanabhuti, Atze Jan van der Goot

**Affiliations:** a Food Process Engineering, Wageningen University & Research, PO Box 17, 6700 AA, Wageningen, the Netherlands; b Division of Food, Nutrition, and Exercise Sciences, University of Missouri, Columbia, MO, 65211, USA

**Keywords:** Meat analogues, Hydrocolloids, Salts, Textural properties

## Abstract

Consumer studies suggest that the meat-like texture of plant-based meat alternatives is crucial for the market success of these products. Many meat analogues contain wheat gluten, because it is cost-effective and give rise to nice fibrous structures. However, individuals with celiac disease cannot consume products containing wheat gluten producing a fibrous structure. To provide meat-like textures, different hydrocolloids with appropriate salt concentrations could be used. Therefore, this study investigated the influence of different hydrocolloids, including high acyl gellan gum, low acyl gellan gum, high methoxyl pectin, low methoxyl pectin, and xanthan at 2%, as well as two types of salts (CaCl_2_ and NaCl) at three concentrations (0%, 0.5%, and 1%) on the macrostructure, microstructure, and mechanical properties of plant-based meat alternatives containing only soy protein isolate and without wheat gluten. The addition of hydrocolloids and salts increased the cross-link bonds and structural compactness at the microscopic level and enhanced the fibrous structure at the microscopic level at different extent. These findings provide insight into how the addition of salts and hydrocolloids can effect plant-based meat alternatives without wheat gluten, which have practical implications for the food industry and are important for their success in the market.

## Highlights

• Influence of different hydrocolloids and salts were investigated in meat analogues.
• Hydrocolloids and salts increased cross-links and compactness at microscopic level.
• Hydrocolloids and salts also enhanced the fibrous structure at microscopic level.
• Appropriate salt and hydrocolloid concentration was crucial to textural properties.
• Those could enhance textural properties of products without wheat gluten.

## Graphical abstract

**Figure:**
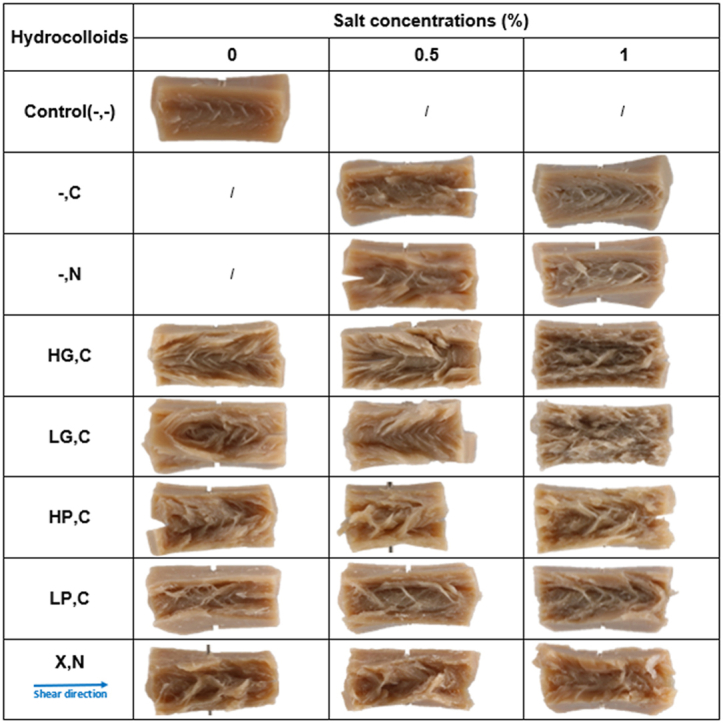

## 1 Introduction

As the global population continues to grow, the demand for protein has reached unprecedented levels, putting significant pressure on the environment through the excessive consumption of meat (D'Silva and Webster, 2017). In addition, the consumption of meat and processed meat products has been linked to several health issues, including obesity, type 2 diabetes, and colorectal cancer (Kołodziejczak et al., 2021). To address these challenges, the food industry has witnessed a surge in interest in developing plant-based meat alternatives, which offer a more sustainable and healthier option (Hoek, 2010).

One critical parameter in the texture and sensory qualities of plant-based meat alternatives is the fibrous structure. Wheat gluten (WG) is a primary ingredient used to create a fibrous structure in plant-based meat alternatives made from various protein sources such as soy or pea, by forming an interconnected network via intermolecular and intramolecular disulfide bonds. Moreover, the inclusion of WG in plant-based meat alternatives can positively impact the texture, binding properties, moisture retention, and sensory attributes of the final product. (Chiang et al., 2021). However, individuals with celiac disease cannot consume plant-based meat alternatives that contain gluten (Theethira and Dennis, 2015).

Hydrocolloids are promising substitutes for WG, given their ability to mimic its functionality and texture. To enhance the functionality of certain hydrocolloids, such as high acyl gellan gum, low acyl gellan gum, high methoxylated pectin, low methoxylated pectin, and xanthan, appropriate salt concentrations are required to produce a network, which can lead to creation of fibrous structure in plant-based meat alternatives (Maltais et al., 2005). Salts, such as sodium chloride and calcium chloride, are often used to improve the sensorial properties of plant-based meat alternatives, such as their taste and texture (Cornet et al., 2021). Moreover, calcium chloride serves as a vital calcium fortifier in food products, influencing hydrophobic interactions and cross-link density as an excellent cross-linking agent (Guo et al., 2019).

The objective of this study is to investigate the influence of different hydrocolloids, including high acyl gellan gum, low acyl gellan gum, high methoxylated pectin, low methoxylated pectin, and xanthan on the creation of fibrous products using SPI as main proteinaceous ingredient. The selection of these hydrocolloids has been made based on their specific characteristics. Xanthan, widely utilized in the meat industry, exhibits gelling, thickening, stabilizing, and emulsifying properties. Gellan gum and pectin, although less commonly used in meat analogues, possess unique attributes that make them suitable for diverse food applications. Gellan gum, renowned for its exceptional gelling abilities, forms robust and stable gels that exhibit resistance to heat and pH variations (Taghian Dinani, Broekema, et al., 2023b). Hydrocolloids like xanthan, gellan gum, and pectin possess thickening and gelling properties, enabling them to create a cohesive and structured network within the protein matrix when combined with appropriate formulations and salt concentrations. Taghian Dinani, Broekema et al. (2023b) investigated xanthan, low acyl gellan gum, and low methylated pectin hydrocolloids in blends containing pea protein isolate (PPI) and WG, while maintaining a fixed concentration of calcium chloride (1%). However, they did not explore the influence of salt in their study. Therefore, this study aims to determine whether salt addition can enhance the textural properties and fibrous structure of plant-based meat alternatives, and how the type and concentration of salt used can modulate the gel and network structure formation such as its mechanical properties (Maltais et al., 2005). Furthermore, this study aims to examine and compare gellan gum with varying degrees of acylation (high and low gellan gum) and pectin with different levels of methylation (low and high methylated pectin). The hypothesis is that type of salt and salt concentration can affect the formation of fibrous structures in plant-based meat alternatives based on blends of SPI and these various hydrocolloids (Grabowska et al., 2014). In conclusion, this study seeks to provide insights into how the addition of salt can affect the textural properties and fibrous structure of plant-based meat alternatives, which can have significant implications for the development of more sustainable and healthier food products.

## 2 Materials and methods

### 2.1 Materials

The soy protein isolate (SPI) used in this study was the SUPRO® 500E IP sourced from Solae (IFF, MO, USA), with a minimum protein content of 90 wt% (N × 6.25). Calcium chloride (CaCl_2_) was procured from Merck (Darmstadt, Germany), while Sigma-Aldrich (Darmstadt, Germany) supplied the sodium chloride (NaCl) (STBK3732). The hydrocolloids employed, high acyl gellan gum (KELCOGEL® LT100), low acyl gellan gum (KELCOGEL® F), high methoxyl pectin (GENU® pectin type YM-115-H), low methoxyl pectin (GENU® pectin type LM-5 CS), and xanthan (KELTROL® F) were all obtained from CP Kelco (Lille Skensved, Denmark). Rhodamine B, used for confocal laser scanning microscopy staining, was procured from Sigma-Aldrich (Zwijndrecht, the Netherlands). The glutaraldehyde solution used in the study, with a 25% glutaraldehyde concentration, was also supplied by Sigma-Aldrich (Zwijndrecht, the Netherlands), and the ethanol (96% v/v) was obtained from VWR International S.A.S. (Rosny-sous-Bois, France).

### 2.2 Methods

#### 2.2.1 Sample preparation

**Fig. 1:**
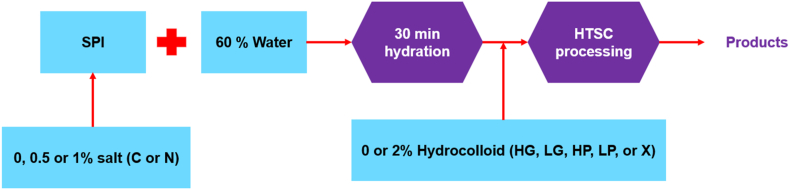
An schematic overview of the preparation of different products. In this overview, SPI represents soy protein isolate, and C, N, HG, LG, HP, LP, and X correspond to CaCl_2_, NaCl, high acyl gellan gum, low acyl gellan gum, high methoxyl pectin, low methoxyl pectin, and xanthan, respectively.

The dry ingredients of all protein blends were kept at a total of 40 wt%. Table 1 shows the formulation of the various blends. Each product was prepared and independently analyzed three times, unless otherwise stated. The preparation of these products is shown in Fig. 1 and is further described in the following four sections.

**Table 1:** An overview of the formulation for protein blends containing 40% dry ingredients. The table includes abbreviations for the additives used in the blends, where SPI represents soy protein isolate, and C, N, HG, LG, HP, LP, and X correspond to CaCl_2_, NaCl, high acyl gellan gum, low acyl gellan gum, high methoxyl pectin, low methoxyl pectin, and xanthan, respectively.

Abbreviations	Dry ingredients (40 wt%)			Water
	Hydrocolloids	Salt	SPI	
**Control (-,-)**	–	–	40%	60%
**-,C(0.5%)**	–	0.5% C	39.5%	60%
**-,C(1%)**	–	1% C	39%	60%
**-,N(0.5%)**	–	0.5% N	39.5%	60%
**-,N(1%)**	–	1% N	39%	60%
**HG,C(0%)**	2% HG	–	38%	60%
**HG,C(0.5%)**	2% HG	0.5% C	37.5%	60%
**HG,C(1%)**	2% HG	1% C	37%	60%
**LG,C(0%)**	2% LG	–	38%	60%
**LG,C(0.5%)**	2% LG	0.5% C	37.5%	60%
**LG,C(1%)**	2% LG	1% C	37%	60%
**HP,C(0%)**	2% HP	–	38%	60%
**HP,C(0.5%)**	2% HP	0.5% C	37.5%	60%
**HP,C(1%)**	2% HP	1% C	37%	60%
**LP,C(0%)**	2% LP	–	38%	60%
**LP,C(0.5%)**	2% LP	0.5% C	37.5%	60%
**LP,C(1%)**	2% LP	1% C	37%	60%
**X,N(0%)**	2% X	–	38%	60%
**X,N(0,5%)**	2% X	0.5% N	37.5%	60%
**X,N(1%)**	2% X	1% N	37%	60%

##### 2.2.1.1 Preparation of control sample

The general procedure for preparing protein blends from Taghian Dinani, Broekema et al. (2023b) was utilized, with slight modifications. Fig. 1 shows that SPI (40%) was mixed with distilled water (60%) using a spatula for approximately 1 min until a homogeneous mixture was obtained. The blended protein mixture was then covered with parafilm to prevent water evaporation and left to hydrate at room temperature for 30 min. Finally, the protein blend was processed in the high-temperature shear cell (HTSC).

##### 2.2.1.2 Preparation of protein blends with salt

To prepare protein blends containing salt, two different proportions of SPI and either calcium chloride (C) or sodium chloride (N) were used. Samples with 0.5% salt contained 39.5% SPI, while those with 1% salt contained 39% SPI, resulting in 40% dry ingredients in all formulations. First, the SPI was mixed with the salt, and the mixture was then added to a beaker with 60% distilled water. The ingredients were mixed with a spatula for approximately 1 min to ensure homogeneity. The resulting protein blend was covered with parafilm to prevent water evaporation and left to hydrate at room temperature for 30 min. Finally, the blend was processed in the HTSC (Fig. 1).

##### 2.2.1.3 Preparation of protein blends with hydrocolloids

To prepare protein blends containing hydrocolloids, 38% SPI was mixed with 60% distilled water in a beaker and stirred with a spatula for approximately 1 min. The resulting mixture was then covered with parafilm and left to hydrate at room temperature for 30 min. Next, 2% hydrocolloid was added to the hydrated protein blend, and the mixture was stirred for about 30 s. In a study conducted by Dekkers et al. (2016), it was reported that the sequential process of hydrating proteins followed by hydrocolloid incorporation resulted in the formation of prominent fibrous structures. Therefore, we employed this method in our study. By adding hydrocolloids after protein hydration, the proteins had already formed a hydrated and interconnected matrix. The subsequent addition of hydrocolloids can facilitate their dispersion throughout the matrix, promoting the formation of a fibrous structure. The resulting protein blend was then processed in the HTSC (Fig. 1).

##### 2.2.1.4 Preparation of protein blends with salt and hydrocolloids

These products contained protein blends along with 2% hydrocolloid (HG, LG, HP, LP, and X) and salt (either CaCl_2_ or NaCl). Two different proportions of SPI and salt were used, depending on the formulation: 37.5% SPI with 0.5% salt and 2% hydrocolloid, or 37% SPI with 1% salt and 2% hydrocolloid. First, the SPI and salt were mixed together in the appropriate ratio. The mixture was then added to a beaker containing 60% distilled water and stirred with a spatula for approximately 1 min to ensure even mixing. The resulting protein blend was covered with parafilm to prevent water evaporation and left to hydrate at room temperature for 30 min. Next, the desired hydrocolloid was added to the hydrated protein blend (at 2% concentration) and stirred for around 30 s. Finally, the protein blend was processed in the HTSC (Fig. 1).

#### 2.2.2 High-temperature shear cell processing protein blends

A high-temperature shear cell (HTSC) from Wageningen University in the Netherlands was utilized to transform protein blends into plant-based meat alternatives. The HTSC processing method, as described by Schreuders et al. (2022), was employed with some modifications. The shear cell was preheated to 120 °C, and the protein blend was introduced into the HTSC and subjected to a constant shearing rate (30 rpm) at 120 °C for 15 min. Subsequently, the sample was cooled to 25 °C using the cooling system (JULABO LH-46, Germany) without a shearing rate (0 rpm) for 10 min inside the shear cell. The products were then extracted and kept in a sealed Ziplock bag at room temperature for at least 1 h before further measurements were taken. For fresh products, fibrous structure, refractive digital microscopy, and tensile strength tests were performed. The samples then were frozen at −18 °C for further tests. Finally, the CLSM and SEM were conducted on frozen products.

#### 2.2.3 Visual observation for fibrous structure

**Fig. 2:**
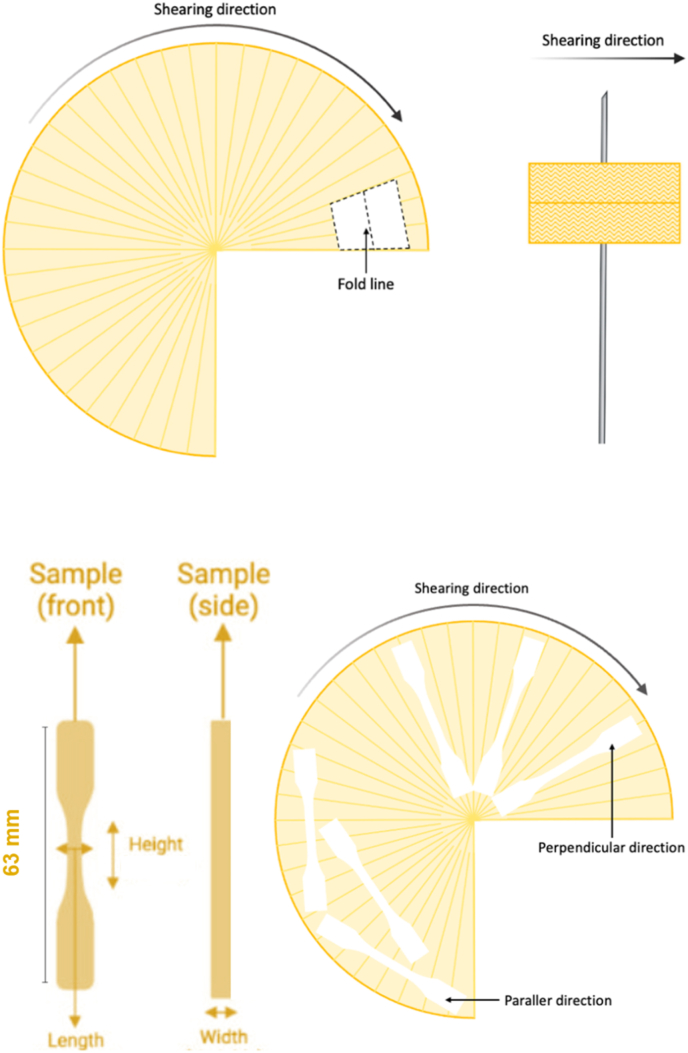
**A)** Visualization of the location where the visual observation for fibrous structure was conducted and **B)** schematic illustration of the bone-shaped bar used for the tensile strength test (left), and visualization of positions of the bars cut out from the products in the parallel and perpendicular directions to the shearing flow direction (right).

To analyze the fibrous structure of the sheared product, a piece was manually cut out and bent along the parallel direction to the shearing flow, as illustrated in Fig. 2A. The piece was then mounted on a long needle and placed in a white mini photo studio with three adjustable light sources (Falcon Eyes DV-80SL, China) from the left, right, and top directions, respectively, to capture images of its fibrous structure using a Canon camera (Japan) (Taghian Dinani, Allaire, et al., 2023a).

#### 2.2.4 Refractive light microscopy

In order to examine the fibrous structure of the fresh product, a refractive light microscope (Smartzoom 5, ZEISS, USA) with 34x magnification was also utilized. The product pieces were prepared in the same manner as the visual observation for fibrous structure in the previous section, following the procedure outlined by Schreuders et al. (2022) with some modifications. Pictures were captured with a resolution of 6.6325 μm/Pixel, utilizing an overview illumination type.

#### 2.2.5 Scanning electron microscopy

Scanning electron microscopy (SEM) was performed following the procedure outlined by Dekkers et al. (2016) and Taghian Dinani, Broekema, et al. (2023b) to acquire information on the microstructure (∼5 μm) of plant-based meat alternatives. Frozen products were cut into rectangular samples measuring 5 mm × 13 mm perpendicular to the shearing direction. Each sample was then placed in a test tube with 20 mL of 2.5% (v/v) glutaraldehyde and gently rotated for 8 h using a Mini Rocker-Shaker (MR1, Riga, Latvia). The sample was then rinsed with demineralized water and placed in another test tube with 20 mL demineralized water and gently rotated overnight with a Mini Rocker-Shaker. The sample was then immersed in a series of ethanol solutions (10, 30, 50, 70, 80, 90 and 100% (v/v)) for at least 1 h per solution. Samples were dried using critical point drying (CPD 300, Leica, Vienna, Austria) and then carefully fractured parallel to the shearing direction. Finally, the samples were coated with a 12 nm thick layer of wolfram (SCD 500, Leica, Vienna, Austria) and evaluated using a field emission scanning electron microscope (Magellan 400, FEI, Eindhoven, the Netherlands) at magnification of 10,000x with a working distance of 6–8 mm and secondary electron detection of 13 pA and 2 kV.

#### 2.2.6 Confocal laser scanning microscopy

The microstructure of the plant based meat alternative products was examined using a Confocal Scanning Laser Microscope (CLSM) (Zeiss type 510, Oberkochen, Germany) according to the procedure described by Taghian Dinani, Broekema et al. (2023b) and Grabowska et al. (2016). Frozen products were cut into samples with dimensions of 3 mm × 8 mm × 10 mm parallel to the shear direction. The samples were then glued onto a metal holder using Cryo compound (Immunologic a WellMed, Duiven, the Netherlands) and sliced into smooth, 60 μm-thick sections at −16 °C using a cryo-microtome (Micron CR50-H, ADAMAS-instruments corp, Rhenen, the Netherlands). The produced slices were placed on a glass slide, stained with Rhodamine B (0.002%), and covered with a cover glass. The stained samples were stored in a dark, humid container at room temperature for at least 1 h to allow absorption of Rhodamine B and prevent moisture loss. Images were taken using a 543 nm HeNe laser, a 405 nm Blue/Violet diode laser, and a 20x EC Plan-Neofluar/0.5 A lens. The analysis was performed using LAS X (Leica Microsystems CMS GmbH, Amsterdam, the Netherlands).

#### 2.2.7 Tensile strength analyses

To analyze the tensile strength of the fresh plant-based meat alternatives, a Texture Analyzer (TA-XTplus Connect, United Kingdom) was used with a static load cell of 100 N, following the procedure outlined by Taghian Dinani, Charles Carrillo et al. (2023c), Taghian Dinani, van der Harst et al. (2023d) and Schreuders et al. (2019). Tensile bars were cut from the fresh product in two directions, parallel and perpendicular to the shearing direction, using a dog bone-shaped mold with a total height of 63 mm (see Fig. 2B). The two ends of each tensile bar were secured in clamps to prevent slippage. The tensile stress (σ, measured in N/m^2^) and tensile strain (ε, unitless) were calculated using the values obtained at the breakpoint, as per the material resistance equations established by Taghian Dinani, van der Harst et al. (2023d). The Young's modulus (E, Pa) was calculated from the linear part of the tensile stress versus tensile strain curve. Then, the anisotropic index of the tensile stress (AI_stress_) was calculated using Equation (1):

(1) $${AI}_{stress}=\frac{\sigma_{paraller}}{\sigma_{perpendicular}}$$

The three tensile bars were tested in both parallel and perpendicular directions for each product and the average results were obtained. The standard deviations were determined based on the variation between the three products in each direction.

### 2.3 Statistics

In the present study, the Complete Random Design (CRD) was used to compare the processing of products with different types and concentrations of salt, as well as with different hydrocolloids. Data analysis was performed using the IBM SPSS software Version 28.0 (New York, USA). To evaluate the statistical significance between samples, a univariate general linear Duncan's test was performed at a significant level of 95% (p ≤ 0.05). The results were reported as mean ± standard deviation (SD). Unless stated otherwise, the experiments were conducted in triplicate.

## 3 Results and discussion

### 3.1 Fibrous structure visual observation

To examine the fibrous structure of various plant-based products produced in the HTSC, a visual analysis was conducted by folding them parallel to the shear flow direction. In Table 2, the Control(-,-) had a uniform gel structure without discernible fibers, which is consistent with previous research by Grabowska et al. (2016), who also found isotropic products when processing SPI-only. When 0.5% salt was added to SPI blends without hydrocolloids (-,C(0.5%) and -,N(0.5%)), products were formed comprising small and thin fibers, while the addition of 1% salt (-,C(1%) and -,N(1%)) led to the formation of intermediate fibers. In meat analogues, the term of intermediate fibers refers to fibrous components that have lengths between short fibers (a few millimeters to a centimeter) and long fibers (several centimeters or more). The fact that these product structures were created could be possibly attributed to the fact that protein isolates can be considered as multi-protein ingredients with a small amount of carbohydrates that come from fibers and could serve as the dispersed phase.

**Table 2:**
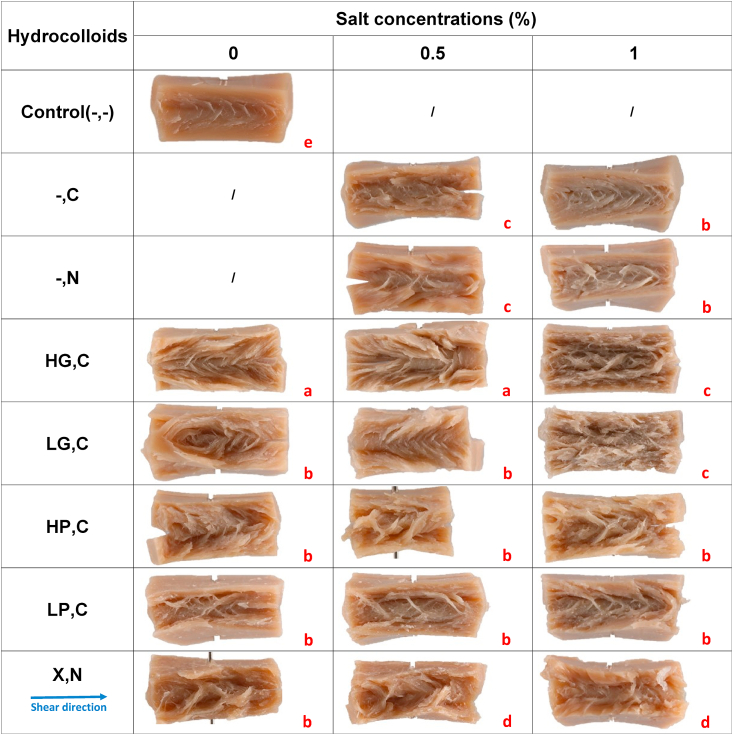
Visual fibrous structure of different plant-based meat alternatives. These products include products without any hydrocolloids (Control(-,-)) and products containing high acyl gellan gum (HG), low acyl gellan gum (LG), high methoxyl pectin (HP), low methoxyl pectin (LP), and xanthan (X) combined with either CaCl_2_ (C) or NaCl (N) at concentrations of 0%, 0.5%, and 1%. The red letters used in this table indicate the type of visual structure that was formed. Specifically, red letters of "a" represents the formation of large fibers, "b" represents the formation of intermediate fibers, "c" represents the formation of thin and small fibrils, and "d" represents a lumpy-like structure and "e" represents a homogenous gel.

The addition of all the mentioned hydrocolloids to SPI blends without salt significantly influenced the structure of the products after heat and shear treatments. This observation confirms the crucial role of hydrocolloids in plant in making fibrous products. Specifically, the addition of HG to SPI blends without salt (HG,C(0%)) and with 0.5% CaCl_2_ (HG,C(0.5%)) resulted in the formation of large fibers, leading to more pronounced fibrous structures than the Control(-,-) and blends with other hydrocolloids. These large fibers indicated a strong adhesion between SPI and the hydrocolloid phase. HG is a high molecular weight hydrocolloid that can form an extensive network, resulting in strong adhesion with the protein (Cornet et al., 2022; Milani et al., 2012). Generally, under shear and heat flows, the hydrocolloids were elongated and oriented in the direction of shear flow, resulting in adhesion between the protein and the hydrocolloids in the HTSC (Schreuders et al., 2022). In fact, the theory of phase separation can explain the formation of fibers in the SPI and hydrocolloid system. Moreover, the addition of LG, HP, LP, and X to SPI blends without salt resulted in the formation of intermediate fibers. In this study, hydrocolloids were used at a low concentration of 2%, were thus expected to act as the dispersed phase, while the SPI blends served as the continuous phase, resulting in the entrapment of the hydrocolloids (Dekkers, Emin, et al., 2018b; Dekkers, Hamoen, et al., 2018c).

Salt addition altered the effect of hydrocolloid addition. Salt addition up to 1% within the HG,C and LG,C groups, resulted in thinner and smaller the fibers. This observation could be attributed to the fact that salt ions can react with the charged sites of protein, enhancing protein solubility and interaction with carbohydrates, resulting in the formation of a more interconnected fibrous structure, and thus less phase separated (Braga et al., 2006; Z. Zhang et al., 2022).

In the X,N group, the incorporation of 0.5% and 1% NaCl led to the formation of lumpy-like structures. The larger fibers observed in X,N(0%) are likely attributable to the use of X as a thickening agent, and the addition of NaCl resulted in only slight differences in the macrostructure. Consequently, varying the types of hydrocolloids and salt concentrations used could yield products with a diversity in textural and fibrous structures. Our findings revealed that the concentration of salt is one of the determining factors in the formation of fibrous structures, though its effect depends both on the hydrocolloid and salt type employed. Our results further suggest that the influence of calcium ions on the gel strength of SPI in section 3.5, with or without hydrocolloids, is more significant than that of sodium ions at equivalent concentrations. This could be attributed to the capacity of calcium ions to intensify the hydrophobic effect of the protein, thereby enabling the formation of calcium bridges that enhance protein aggregation and conformation. In contrast, sodium ions lack the ability to form such bridges (Wang et al., 2018).

### 3.2 Refractive light microscopy

Table 3 presents the fibrous structure of folded plant-based meat substitutes, assessed using refractive light microscopy. The Control(-,-) sample displayed a homogenous gel structure without visible fibers, consistent with the observation in Table 2. The incorporation of hydrocolloids had a positive impact on fibrous structures, as shown in Table 3 at this magnification. All treatments containing hydrocolloids and lacking salt (HG,C(0%), LG,C(0%), HP,C(0%), LP,C(0%), and X,N(0%)) exhibited a fibrous structure. This indicates again that hydrocolloids acted as the disperse phase in protein blends, leading to the formation of fibrous structures at a magnification of 34x (Dekkers, Boom, et al., 2018a). Fiber formation takes place when droplets of the dispersed phase become trapped within the continuous phase and undergo deformation aligned with the shear flow. This deformation progressively leads to the emergence of elongated domains, ultimately forming a structure resembling fibers. Given the study's utilization of a low concentration of hydrocolloid (2%) and a high concentration of SPI (minimum 37%), it is expected that the hydrocolloid functions as the dispersed phase (Dekkers, Hamoen, et al., 2018c; Taghian Dinani, Broekema, et al., 2023b). Furthermore, the addition of 1% CaCl_2_ to SPI blends with HG and LG, HP, and LP resulted in thinner fibers compared to the same products without salt. Conversely, the addition of NaCl to SPI blends with X had a negligible effect on the fibrous structure, resulting in limited differences in macrostructure in Table 3. These observations can be attributed to the distinct mechanisms of structure formation. Gellan gum and pectin acted as gelling agents, though they differ in their mechanisms. Gellan gum can form a stacked double helix type of junction zone with cations (Morris et al., 1996, 2012; Sworn and Stouby, 2021), whereas pectin can form the egg-box model and is stabilized by electrostatic integrations by cations (Einhorn-Stoll et al., 2021; Gawkowska et al., 2018). Therefore, the addition of CaCl_2_ to SPI blends with these hydrocolloids resulted in gelation for both gellan gum and pectin, enhancing the stabilization of hydrocolloids and leading to an increase in the number of thin fibrils. It is worth mentioning that HG led to more interconnected and thinner fibers in the products in comparison to LG confirming more effective role of a stacked double helix type of junction zone with cations in production of fibrous structure. However, xanthan was employed as a thickening agent (Saha and Bhattacharya, 2010), and the increasing concentration of NaCl only induced the viscosity changes, resulting in limited differences in macrostructure (Braga et al., 2006). Upon comparing Table 2, Table 3 and it is evident that the differences between the hydrocolloids are less pronounced at higher magnification levels in Table 3 than in Table 2. Nonetheless, the effect of salt became more conspicuous at higher magnification levels in Table 3. It is important to note that the impact of salt concentration may depend on the type of hydrocolloid used in Table 3. For instance, CaCl_2_ had a greater effect on SPI blends containing HG and LG, as opposed to those containing HP and LP.

**Table 3:**
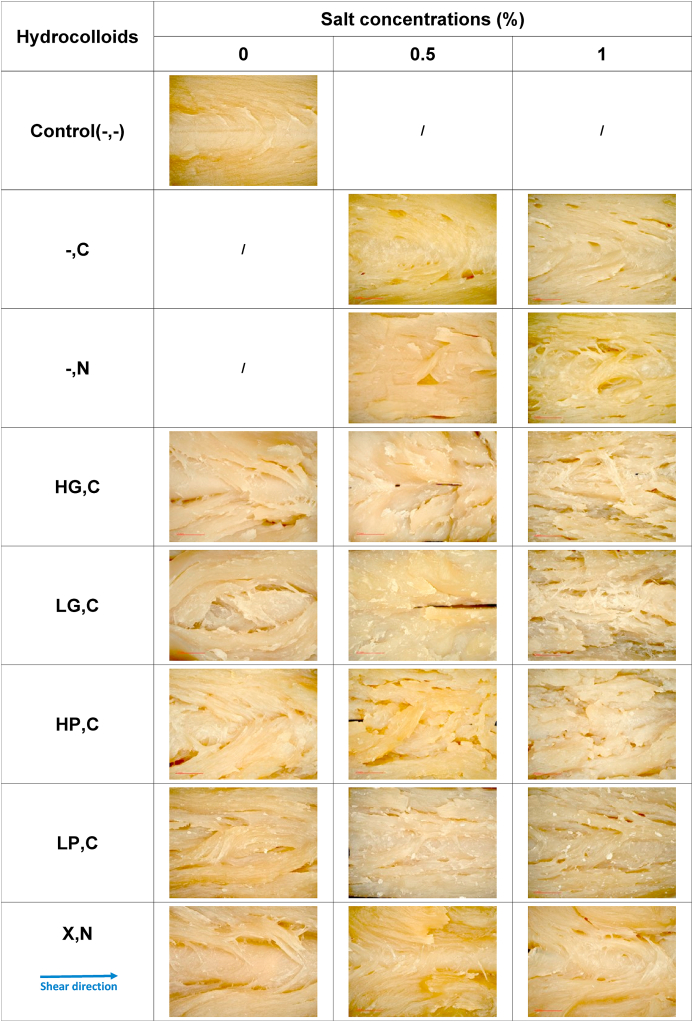
Refractive light microscopic figures of the folded plant-based meat alternatives without any hydrocolloids (Control(-,-)) and products containing high acyl gellan gum (HG), low acyl gellan gum (LG), high methoxyl pectin (HP), low methoxyl pectin (LP), and xanthan (X), combined with either CaCl_2_ (C) or NaCl (N) at concentrations of 0%, 0.5%, and 1%.

#### 3.2.1 Scanning electron microscopy

Scanning electron microscopy (SEM) pictures at magnifications of 10,000 times were used to investigate the microstructural characteristics of plant-based meat alternatives. In Table 4, the Control(-,-) product showed a needle and cluster structure without visible filaments in the shear flow direction. However, the addition of 1% NaCl resulted in a similar but more compact needle and cluster structure compared to Control(-,-). The addition of 1% CaCl_2_ further increased the compactness of the SPI compared to Control(-,-). This observation was consistent with the visual fibrous structure (Table 2) and refractive microscopy pictures (Table 3), suggesting that a compact structure could result in some small and thin interconnected fibrous structure.

**Table 4:**
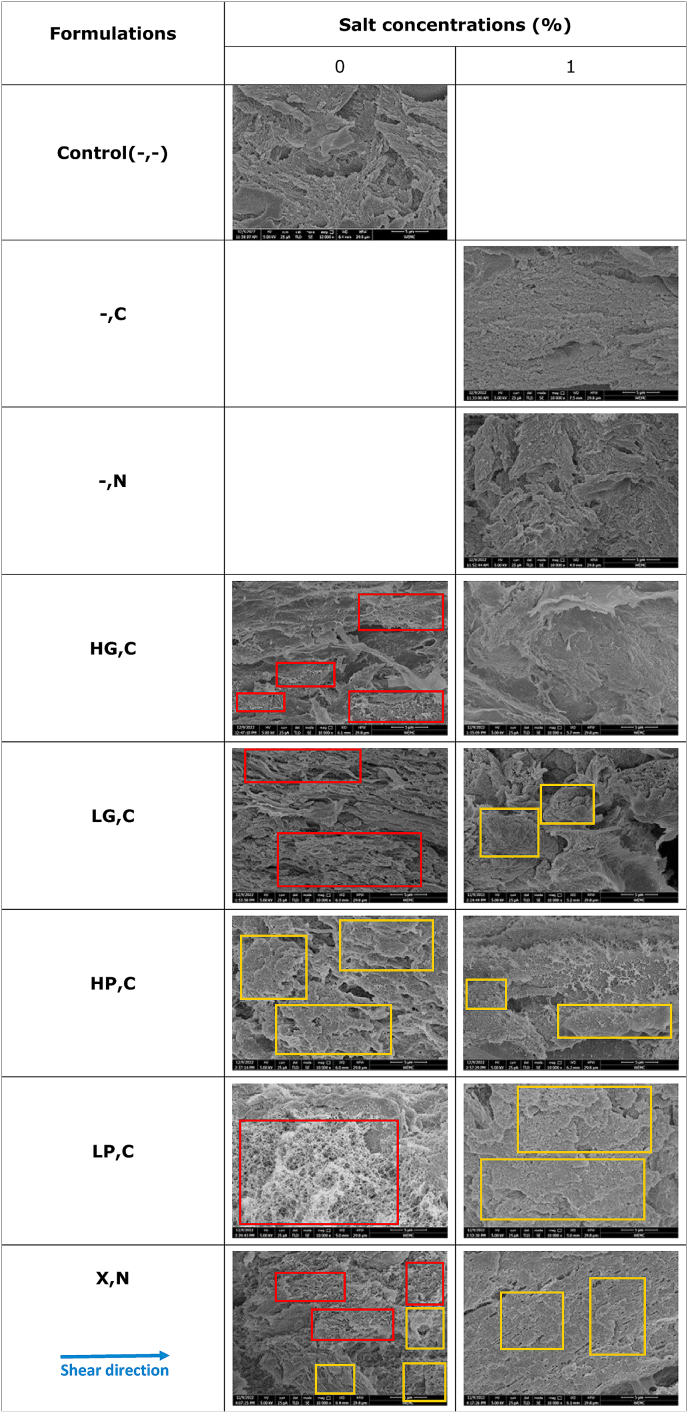
Overview of SEM figures of plant-based meat alternatives without any hydrocolloids (Control(-,-)) and products containing high acyl gellan gum (HG), low acyl gellan gum (LG), high methoxyl pectin (HP), low methoxyl pectin (LP), and xanthan (X), combined with either CaCl_2_ (C) or NaCl (N) at concentrations of 0%, 0.5%, and 1%. In this table, red boxes indicated the spider web morphology, and yellow boxes indicated the cauliflower-like morphology.

The addition of HG and LG to SPI blends without salt (HG,C(0%) and LG,C(0%)) resulted in pronounced filaments with elongated orientation. In Table 4, HG,C(0%), LG,C(0%), and LP,C(0%), showed spiderweb morphology with varying extent, while HP,C showed a clear cauliflower morphology. LP,C(0%) exhibited the most pronounced spiderweb morphology in comparison to other samples in this table. X,C(0%) showed the combination of both spider web and cauliflower morphologies. Conversely, the addition of HP to SPI blends without salt resulted in a cauliflower-like morphology. Globular proteins typically form cauliflower-like and spiderweb morphologies for gel formation. The cauliflower-like morphology was associated with the packed globular domains, while the spiderweb morphology was related to the string-like domains (Grabowska et al., 2014). Moreover, the spiderweb could be linked to the entrapment of air bubbles (Dekkers, Hamoen, et al., 2018c).

The addition of 1% salt (CaCl_2_ or NaCl) had a significant impact on the microlevel structure of the products. The presence of salt led to a more compact structure in all samples with and without hydrocolloids. For instance, the spiderweb morphology of LP,C(0%) transformed into a compact cauliflower-like structure in LP,C(1%) after the addition of 1% CaCl_2_. Furthermore, X,N(1%) displayed a coarse and sandy surface with a compacter structure than that in X,N(0%). Additionally, the incorporation of 1% CaCl_2_ into SPI blends along with HG, LG, and LP resulted in the formation of a cauliflower-like morphology. This phenomenon is believed to be due to the salting out effect, where the high concentration of salt reduces protein solubility, leading to protein aggregation (Z. Zhang et al., 2022). Similarly, Wang et al. (2018) investigated the impact of different salt ions on the gel properties of mixed gels containing Mesona blumespolysaccharide and SPI and found that the presence of calcium and sodium ions resulted in a more uniform, smaller pore size, smoother surface, and compact structure in the mixed gels, which is consistent with our findings. Furthermore, their study also reported that calcium ions promoted better gel microstructure than sodium ions due to the stronger interactions between denatured protein molecules that lead to protein aggregation and the formation of an orderly dense gel network, particularly at low ionic strength (Wang et al., 2018). Therefore, the protein gel formation and network structure can be affected by increasing ionic strength due to the addition of salts (Braga et al., 2006).

#### 3.2.2 Confocal laser scanning microscopy

Confocal Laser Scanning Microscopy (CLSM) was employed to assess the shape and orientation of protein domains in plant-based meat alternatives with various formulations in the direction of shear flow. The results in Table 5 indicate that Control(-,-), -,N(1%), and -,C(1%) showed a lack of elongated orientation. Since no hydrocolloids were added to these treatments, it was assumed that the black shadow in these samples was due to the entrapment of air bubbles or water. The incorporation of hydrocolloids in SPI blends without salt led to elongated protein filaments. For instance, the HG,C(0%) treatment displayed clear elongated domains with elongated black cracks in between. The domains in the HP,C(0%) treatment exhibited less elongation with fewer dark areas than those in the HG,C(0%) treatment. Additionally, the X,N(0%) treatments displayed less elongation but wider black cracks compared to those in the HP,C(0%) treatment. In these pictures, these black cracks could be attributed to the entrapment of air bubbles, water, or hydrocolloids domains. Furthermore, the addition of HP, LP, and X to SPI blends without salt displayed high-intensity red regions (represented by white boxes) in CLSM images compared to Control(-,-). The presence of these regions could be attributed to the high concentration of SPI in this area. The addition of hydrocolloids probably resulted in competition for water between SPI and hydrocolloids during processing, which led to a reduction in the amount of water available for the SPI phase. This caused an increase in protein concentration (Braga et al., 2006). Moreover, hydrocolloids could form more interconnected networks during the thermo-mechanical processing in HTSC (Dekkers, Hamoen, et al., 2018c). This variation in protein concentration could result in variations in fluorescence intensity in the CLSM images.

**Table 5:**
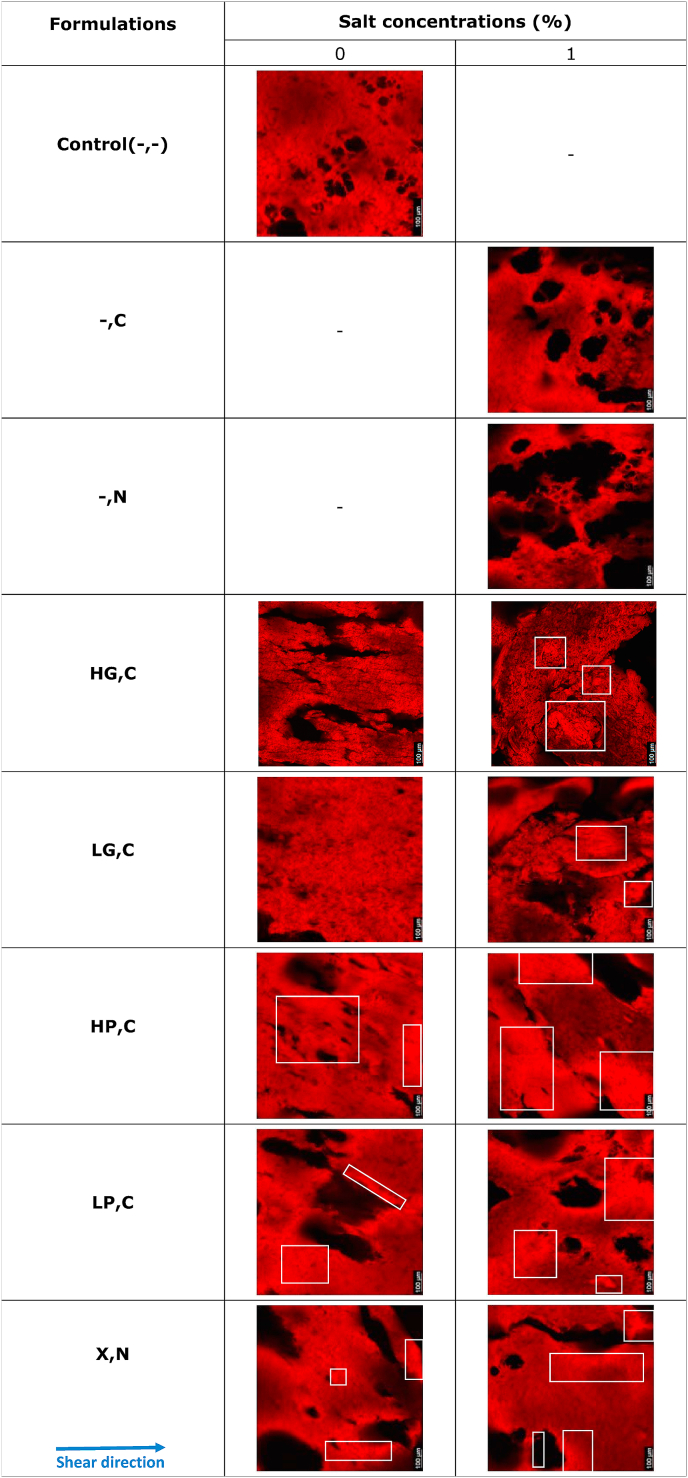
Overview of CLSM figures of plant-based meat alternatives without any hydrocolloid (Control (-,-)) and the products with different hydrocolloids of high acyl gellan gum (HG), low acyl gellan gum (LG), high methoxyl pectin (HP), low methoxyl pectin (LP) and xanthan (X) with two types of salts (CaCl_2_ (C) and NaCl (N)) at three concentrations (0, 0.5, and 1%). In this table, white boxes indicated the high-intensity red regions.

The addition of salt (CaCl_2_ or NaCl) into the SPI and hydrocolloids systems resulted in varied structures that depended on the type of hydrocolloid used. The addition of 1% CaCl_2_ to HG, LG, HP, LP, and 1% NaCl to X resulted in larger high-intensity red regions (represented by white boxes) than similar protein blends without salt. This phenomenon could be attributed to the increasing concentration of salt ions, which could compete with protein for water molecules, resulting in an increase in protein concentrations. This, in turn, could lead to the destruction of the hydration layer on the protein surface, ultimately resulting in the formation of larger protein aggregates. Furthermore, the addition of salt to all the samples with hydrocolloids resulted in similar or more elongated structures in the direction of shear flow.

### 3.3 Mechanical properties

#### 3.3.1 Young's modulus

**Fig. 3:**
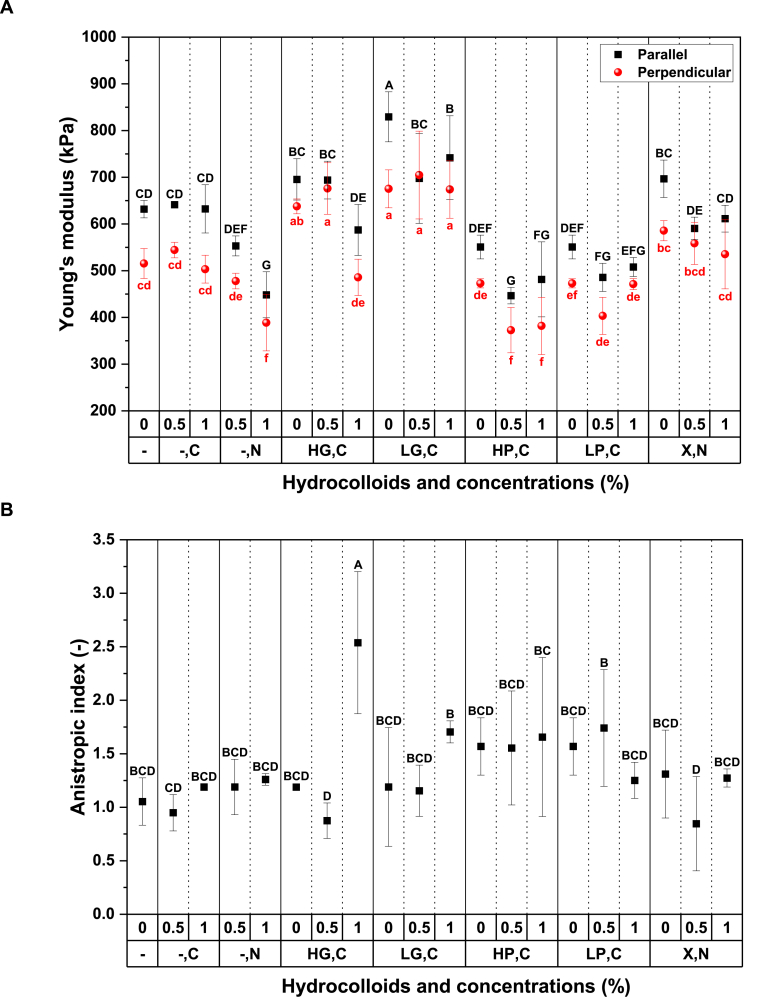
**A)** The Young's modulus values (kPa) and **B)** anisotropic index values of tensile stress of meat analogue without any hydrocolloid (Control (-,-)) and the products with different hydrocolloids of high acyl gellan gum (HG), low acyl gellan gum (LG), high methoxyl pectin (HP), low methoxyl pectin (LP) and xanthan (X) with two types of salts (CaCl_2_ (C) and NaCl (N)) at three concentrations (0, 0.5, and 1%). In figure A, black capital letters display the statistically significant difference of the tensile strain values in parallel direction (p ≤ 0.001), and red lower letters display the statistically significant difference of the tensile strain values in perpendicular direction (p ≤ 0.001). In figure B, various English letters display statistically significant difference of results (p ≤ 0.001). (For interpretation of the references to color in this figure legend, the reader is referred to the Web version of this article.)

The Young's modulus is a measure of a material's stiffness or elasticity (Muster and Prestidge, 2002) and is a small deformation property that provides more information about the linkage of biopolymers such as protein and polysaccharide (Braga et al., 2006). As shown in Fig. 3A, the highest and lowest Young's modulus values in the parallel direction to the shearing flow direction were observed for LG,C(0%) (829.37 ± 53.66 kPa) and HP,C(0.5%) (446.57 ± 17.63 kPa), respectively. Again, in the perpendicular direction, LG,C(0.5%) and HP,C(0.5%) had the highest and lowest Young's modulus values (704.70 ± 93.84 kPa and 372.56 ± 48.37 kPa, respectively). Taghian Dinani, Broekema et al. (2023b) reported that the addition of LG into PPI and WG increased the fibrous structure and tensile strength. Moreover they reported that the addition of LG to PPI and WG led to a larger Young's modulus in both directions compared to other hydrocolloids, including LP and X. The same results were obtained in this study in plant-based meat alternatives even without WG and with the different protein source of SPI. In fact, the hypothesis can be confirmed that adding hydrocolloids could enhance the textural attributes of soy protein-based products without WG, and different kinds of hydrocolloids have various effects on these attributes. For instance, the highest observed increase in Young's modulus of LG,C(0%) sample could be attributed to the fact that LG absorbed more water, making the SPI phase more concentrated.

Generally, Fig. 3A illustrates that the addition of salts to various hydrocolloids resulted in either a reduction or negligible change in the Young's modulus, in both the parallel and perpendicular directions. For instance, in the HG,C group, the addition of 1 wt% CaCl_2_ significantly decreased (p ≤ 0.001) the Young's modulus values in both parallel and perpendicular directions compared to HG,C(0%) and HG,C(0.5%) treatments. Wang et al. (2018) investigated the effect of sodium and calcium ions (0.005, 0.01, 0.015, and 0.02 M) on the gel properties and microstructure of a mixture of Mesona blumes polysaccharide and SPI. They reported that appropriate concentrations of sodium and calcium ions resulted in increased gel strength of the mixture, while higher concentrations of calcium ion (0.02 M) and sodium ions (0.015–0.02 M) reduced gel strength. The excessive addition of sodium or calcium ions could lead to protein aggregation and decreased gel strength. The addition of Ca^2+^ ions can neutralize electrostatic repulsion and form salt bridges between protein aggregates to form a space-filling network. However, high ionic strength can result in a salting out effect or considerable aggregated structure for SPI heat-set gels and play a negative role in gel formation (Maltais et al., 2005). More studies have suggested that salts added to the SPI and hydrocolloid blend can have a dual effect (salting in and salting out effects) based on their concentration on the textural properties of plant-based meat alternatives (Braga et al., 2006; Pan et al., 2017; Z. Zhang et al., 2022).

#### 3.3.2 Anisotropic index

The anisotropic index (AI) based on stress value is a measure of the ratio between tensile stress values in parallel and perpendicular directions. As shown in Fig. 3B, AI stress values ranged from 0.85 ± 0.44 for X,N(0.5%) to 2.54 ± 0.66 for HG,C(1%). The high standard deviation for some treatments was likely due to the heterogeneity of the product material in parallel and perpendicular directions (Jia et al., 2021). The highest and significantly different AI stress value (p ≤ 0.001) of HG,C(1%) was consistent with the visual observation of nice small, thin, and interconnected fibrils of this sample in Table 2, Table 3 and its compact SEM picture in Table 4.

The Control(-,-) sample had a homogeneous structure without fibrils, as shown in Table 2, Table 3, which was consistent with the AI stress value of 1.05 ± 0.22 in Fig. 3B. This suggests that the mechanical properties of Control(-,-) were similar in the parallel and perpendicular directions, indicating an isotropic structure (Grabowska et al., 2014). When comparing Control(-,-) to other treatments, the AI stress values of products containing different hydrocolloids and SPI without salt showed an increasing trend, although there was no significant difference in most products (p > 0.05). However, all hydrocolloids played an important role in the formation of fibrous structure, as seen in the visual observations in Table 2, Table 3 Furthermore, although HG,C(0.5%), LP,C(1%), X,N(0.5%), and X,N(1%) had AI stress values around or less than 1, these products showed varying degrees of fibrous structure in Table 2, Table 3 These observations were inconsistent with the AI stress values in Fig. 3B, indicating that AI values could not be used as a single parameter for the fibrous structure (McClements et al., 2021). This could be due to the alignment of protein phases horizontally or vertically in the shear cell, resulting in fibrous or layered structures, respectively (Grabowska et al., 2014). Both fibrous and layered structures were observed at a macro level, but a layered structure in the products had similar mechanical strength in the parallel and perpendicular directions, resulting in an AI value around 1 (Fig. 3B).

## 4 Conclusion

In this study, the influence of different hydrocolloids (high acyl gellan gum (HG), low acyl gellan gum (LG), high methoxyl pectin (HP), low methoxyl pectin (LP) and xanthan (X) at 2% with two types of salts (CaCl_2_ (C) and NaCl (N)) at three concentrations (0, 0.5, and 1%)) on textural properties of plant-based meat alternatives containing soy protein isolate (SPI) without wheat gluten (WG) processed with the high-temperature shear cell (HTSC) was investigated. HG,C(0%) resulted in the formation of large fibers, while the LG,C(0%), HP,C(0%), LP,C(0%), and X,N(0%) resulted in the formation of intermediate fibers in the macrostructure. On the macroscale, the addition of salt showed limited influence on fibrous structure for LP and X. However, the addition of salt especially at 1% led to more and thinner fibers with different extents in other samples without and with different hydrocolloids. Overall, it can be concluded that the inclusion of these hydrocolloids has the potential to improve the textural properties of plant-based meat alternatives produced using SPI as main ingredients. Besides, the specific type of hydrocolloids played an important role on textural properties. Furthermore, the appropriate concentration of salt added to the SPI and hydrocolloid blends was crucial to textural properties. These findings can be valuable for the development of plant-based meat alternatives with improved texture and sensory characteristics.

## CRediT authorship contribution statement

**Somayeh Taghian Dinani:** Term, Conceptualization, Formal analysis, Validation, Visualization, Supervision, Writing – review & editing. **Yunyu Zhang:** Investigation, Formal analysis, Validation, Visualization, Writing – original draft. **Bongkosh Vardhanabhuti:** Funding acquisition, Writing – review & editing. **Atze Jan van der Goot:** Term, Conceptualization, Supervision, Funding acquisition, Writing – review & editing.

## Declaration of competing interest

No potential conflict of interest was reported by the authors.

## Data Availability

Data will be made available on request.
